# Supplementary material for: Rare Deletions or Large Duplications Contribute to Genetic Variation in Patients with Severe Tinnitus and Meniere Disease
Source: Genes (Basel). 2023 Dec 22;15(1):22. doi: 10.3390/genes15010022 (PMC10815708; doi:10.3390/genes15010022)
Supplement: Supplementary file 1 [file genes-15-00022-s001.zip › R1_genes-2755127_supplementary.pdf]

genes

MDPI

Article

Rare deletions and a large duplication in patients with severe tinnitus and Meniere Disease

Alba Escalera-Balsera[1,2,3], Alberto M. Parra-Perez[1,2,3], Alvaro Gallego-Martinez[1,2,3], Lidia Frejo[1,2,3,4], Juan Martin-Lagos[1,2,5], Victoria Rivero de Jesus[6], Paz Pérez-Vázquez[7], Patricia Perez-Carpena[1,2,3,8], Jose A. Lopez-Escamez[1,2,3,4]*

1 Otology & Neurotology Group CTS495, Instituto de Investigación Biosanitaria, ibs.GRANADA, Universidad de Granada, 18071 Granada, Spain; albaescalera@correo.ugr.es, amparraperez@ugr.es, alvaro.gallego@ugr.es, lidia.frejonavarro@sydney.edu.au
2 Division of Otolaryngology, Department of Surgery, Universidad de Granada, Granada, 18016 Granada, Spain.
3 Sensorineural Pathology Programme, Centro de Investigación Biomédica en Red en Enfermedades Raras, CIBERER, 28029 Madrid, Spain.
4 Meniere's Disease Neuroscience Research Program, Faculty of Medicine & Health, School of Medical Sciences, The Kolling Institute, University of Sydney, 2065 Sydney, New South Wales, Australia; jose.lopezescamez@sydney.edu.au
5 Department of Otorhinolaryngology, Hospital Clinico Universitario San Cecilio, 18016 Granada, Spain; juan.martinlagos.sspa@juntadeandalucia.es
6 Hospital Clínic, Department of Otorhinolaryngology, Barcelona, Spain; vriveroj@csi.cat
7 Servicio de Otorrinolaringología, Hospital Universitario Central de Asturias, 33011 Oviedo, Asturias, Spain; pazperezv@gmail.com
8 Department of Otorhinolaryngology, Hospital Universitario Virgen de las Nieves, 18014 Granada, Spain; percarpena@ugr.es
* Correspondence: jose.lopezescamez@sydney.edu.au

Supplementary Materials

Supplementary Figures

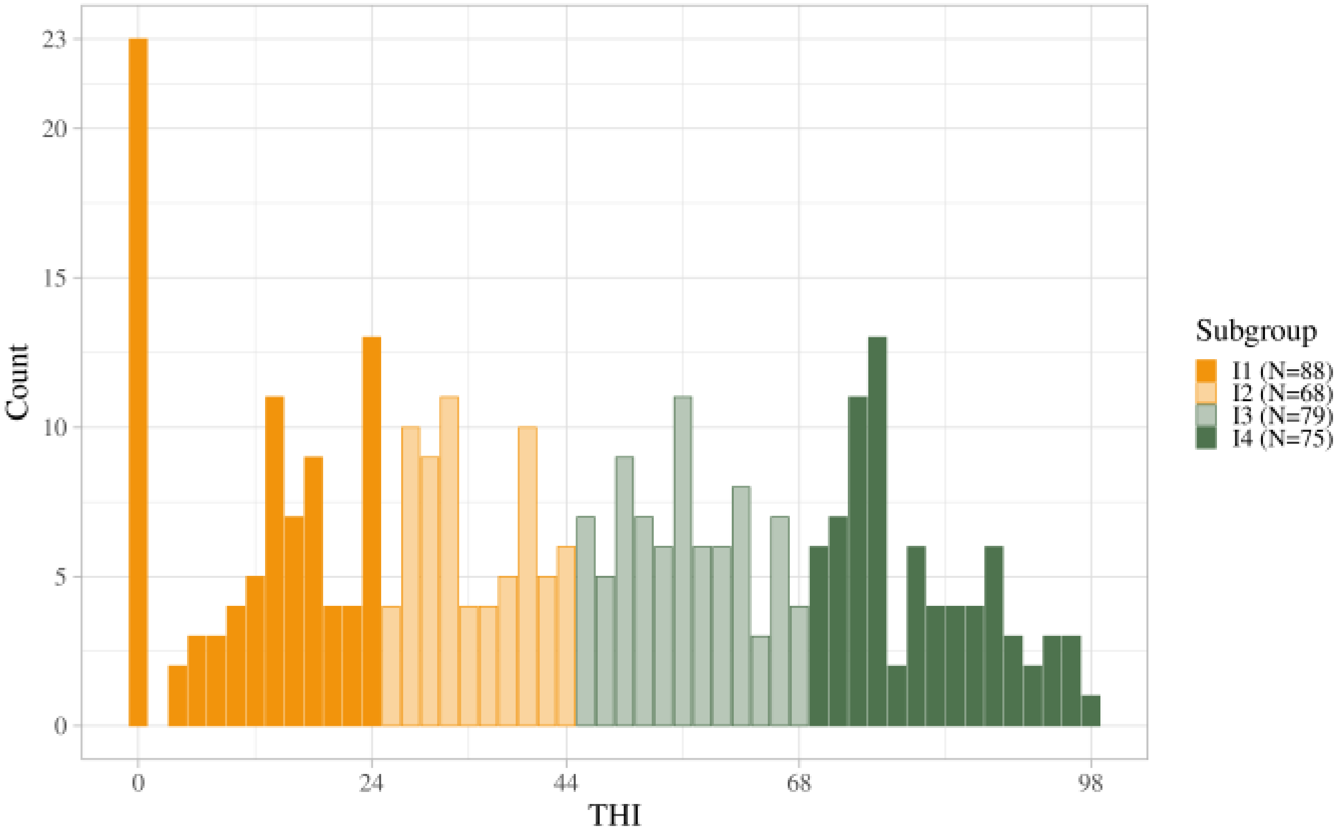

Figure S1. *Distribution of the Tinnitus Handicap Inventory (THI) in the study cohort. The minimum value, quantiles and maximum value are defined in the x-axis; they allow subgrouping the sample in four intervals: I1, I2, I3 and I4.*

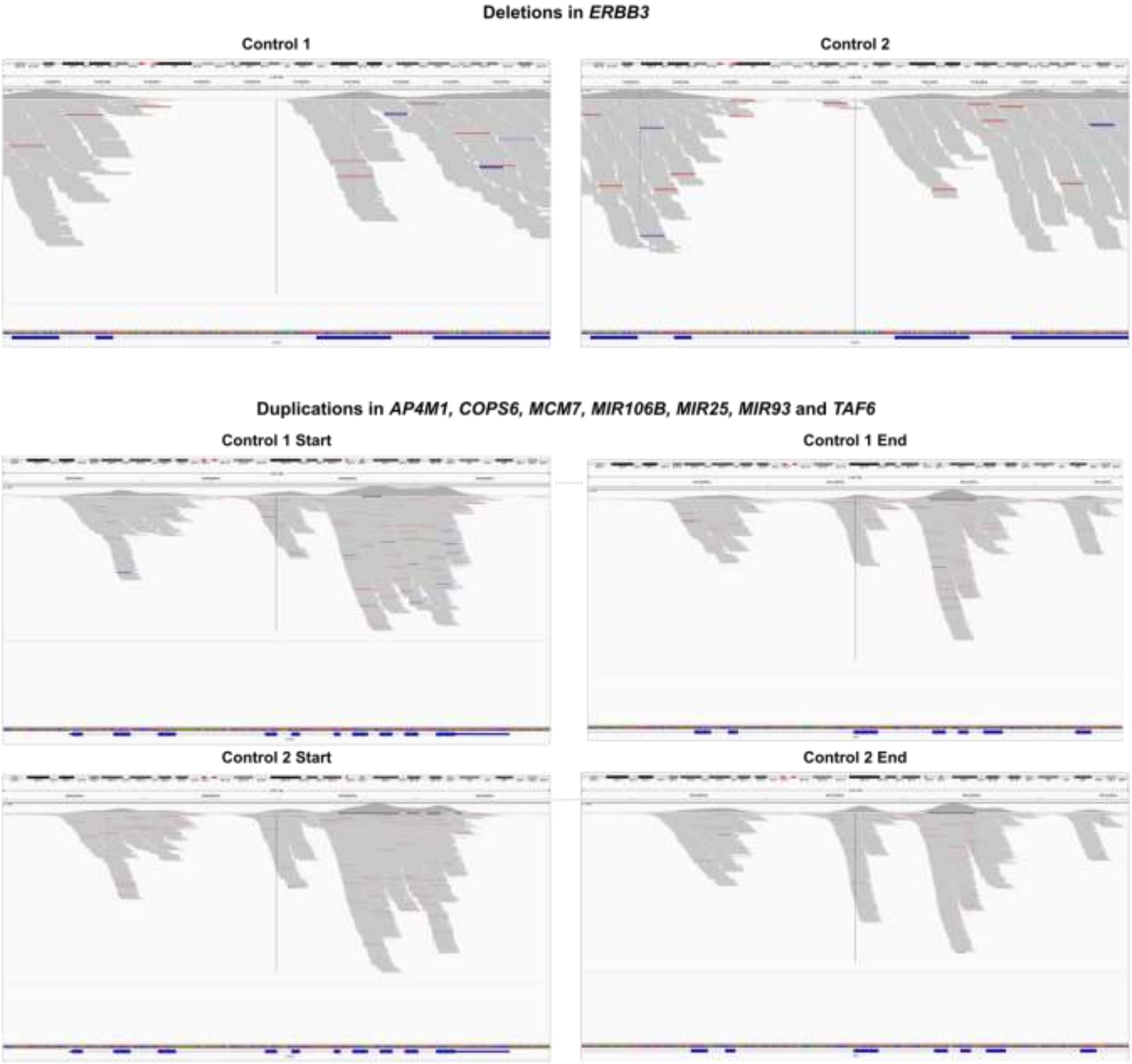

Figure S2. Validation in IGV in two controls without the structural variants (SVs) of the deletions in *ERBB3* gene; and the large duplication in *AP4M1, COPS6, MCM7, MIR106B, MIR25, MIR93* and *TAF6* genes.

Supplementary Tables

Table S1. Summary of structural variants (SV) found in severe tinnitus individuals by TIDDIT.

Chr	Start	End	Length	SV type	Individuals	Gene symbol	ACMG
12	56100031	56100173	142	Duplication	I4-40, I4-41	*ERBB3*	US
12	56100248	56101058	810	Duplication	I4-40, I4-41	*ERBB3*	US
12	56101365	56101527	162	Duplication	I4-40, I4-41	*ERBB3*	US
7	100089054	100112327	23,273	Duplication	I4-28, I4-37	*AP4M1, COPS6, MCM7, MIR106B, MIR25, MIR93, TAF6*	US

Chr: Chromosome; ACMG: American College of Medical Genetics and Genomics; US: Uncertain significance.

Table S2. American College of Medical Genetics and Genomics (ACMG) criteria for the structural variants found in severe tinnitus individuals.

**Variant**	**NC_000012.12:g.56100028_56100172del**	
**Gene(s)**	*ERBB3*	
**Pathogenicity**	Uncertain significance	
**Criteria:**		
**Genomic Content**	Uncertain significance	This structural variant affects 1 domain in 1 protein: 1 x ERBB3_HUMAN reported in UniProt Regions.
**Gene**	Uncertain significance	This structural variant affects 1 coding gene: *ERBB3*.
**Inheritance**	Uncertain significance	No phenotypes or diseases provided.
**Literature**	Benign	Found 1 common variant by DGV, 0 benign CNVs and 0 pathogenic CNVs reported.
**Gene/Regions Overlap**	Uncertain significance	CNV has both break points in a loss-of-function causing gene, but it doesn't contain any known loss-of-function variant.
**Variant**	**NC_000012.12:g.56100243_56101058del**	
**Gene(s)**	*ERBB3*	
**Pathogenicity**	Uncertain significance	
**Criteria:**		
**Genomic Content**	Uncertain significance	This structural variant affects 1 domain in 1 protein: 1 x ERBB3_HUMAN reported in UniProt Regions.
**Gene**	Uncertain significance	This structural variant affects 1 coding gene: *ERBB3*.
**Inheritance**	Uncertain significance	No phenotypes or diseases provided.
**Literature**	Benign	Found 1 common variant by DGV, 0 benign CNVs and 0 pathogenic CNVs reported.
**Gene/Regions Overlap**	Uncertain significance	CNV has both break points in a loss-of-function causing gene, but it doesn't contain any known loss-of-function variant.

**Variant**	**NC_000012.12:g.56101359_56101526del**	
**Gene(s)**	*ERBB3*	
**Pathogenicity**	Uncertain significance	
**Criteria:**		
**Genomic Content**	Uncertain significance	This structural variant doesn't affect any known domain, but affects 2 coding-genes.
**Gene**	Uncertain significance	This structural variant affects 2 coding genes: ENSG00000257411 and *ERBB3*.
**Inheritance**	Uncertain significance	No phenotypes or diseases provided.
**Literature**	Benign	Found 1 common variant by DGV, 0 benign CNVs and 0 pathogenic CNVs reported.
**Gene/Regions Overlap**	Uncertain significance	CNV has both break points in a loss-of-function causing gene, but it doesn't contain any known loss-of-function variant.
**Variant**	**NC_000007.14:g.100089053_100112257dup**	
**Gene(s)**	*AP4M1, COPS6, MCM7, MIR106B, MIR25, MIR93, TAF6*	
**Pathogenicity**	Uncertain significance	
**Criteria:**		
**Genomic Content**	Uncertain significance	This structural variant affects 11 domains in 4 proteins: 5 x MCM7, HUMAN, 3 x TAF6, HUMAN, 2 x CSN6, HUMAN and 1 x AP4M1, HUMAN reported in UniProt Regions.
**Gene**	Uncertain significance	This structural variant affects 4 coding genes: *AP4M1*, *COPS6*, *MCM7* and *TAF6*.
**Inheritance**	Uncertain significance	No phenotypes or diseases provided.
**Literature**	Uncertain significance	Found 0 benign CNVs, 0 common variants by DGV and 0 pathogenic CNVs reported.
**Gene/Regions Overlap**	Uncertain significance	No condition is met.
