# Supplementary figures and images for: Rare Deletions or Large Duplications Contribute to Genetic Variation in Patients with Severe Tinnitus and Meniere Disease
Source: Genes (Basel). 2023 Dec 22;15(1):22. doi: 10.3390/genes15010022 (PMC10815708; doi:10.3390/genes15010022)

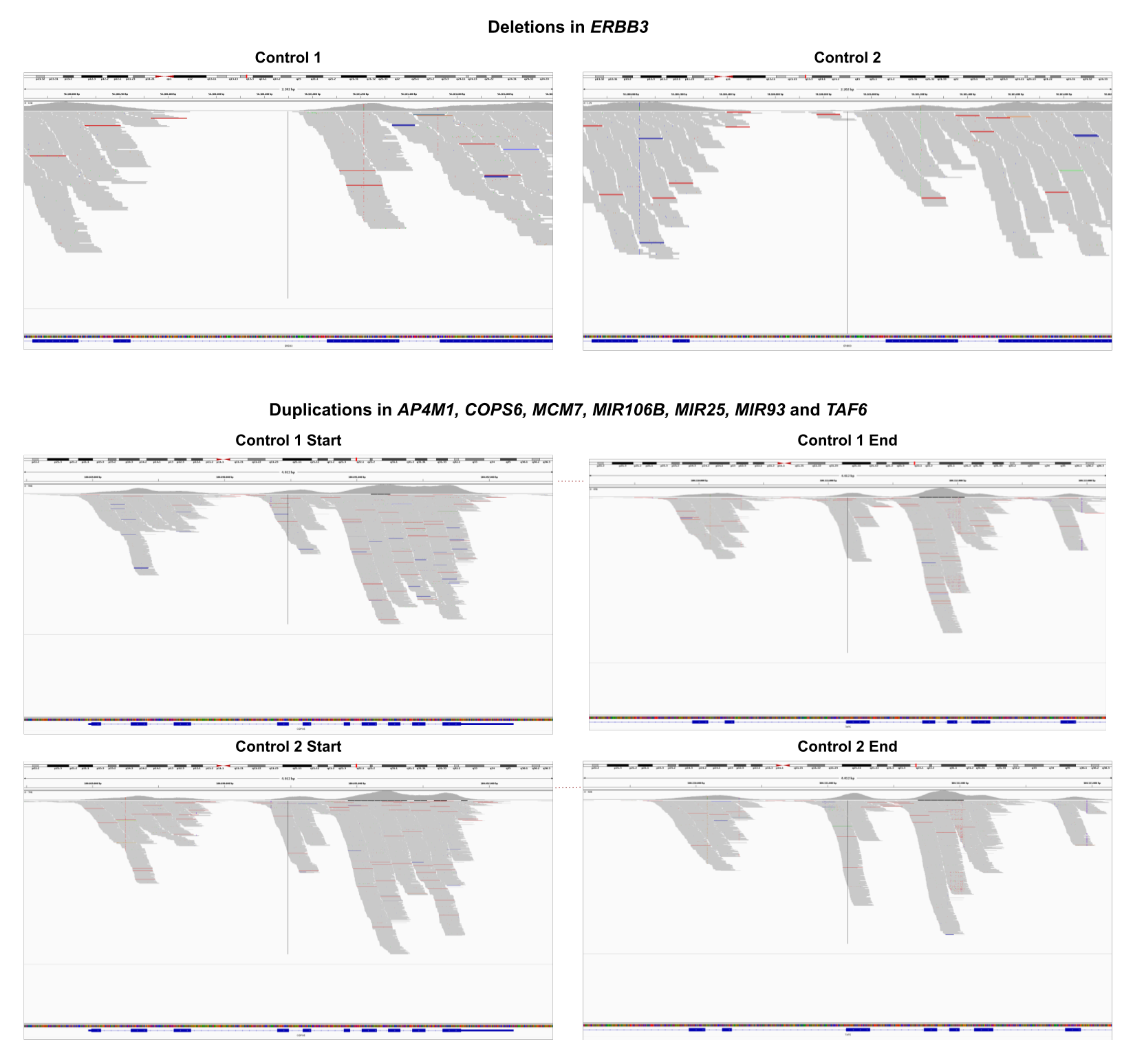

Supplement: Supplementary file 1 [file genes-15-00022-s001.zip › FigureS2.png]

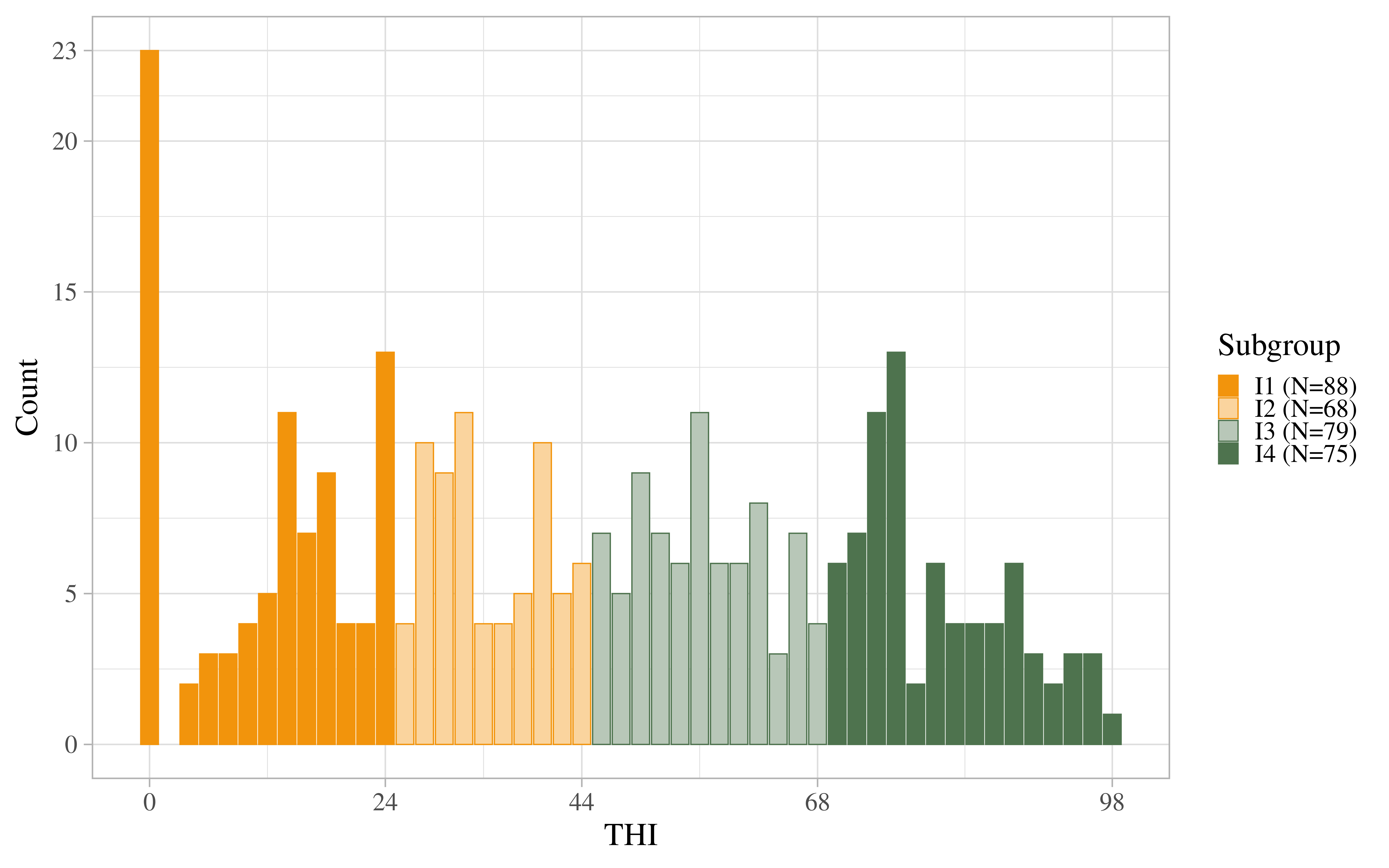

Supplement: Supplementary file 1 [file genes-15-00022-s001.zip › FigureS1.png]
